# Developing and conducting a language inclusivity assessment on a health science library's website, LibGuides, and signage

**DOI:** 10.5195/jmla.2024.1691

**Published:** 2024-01-16

**Authors:** Jane Morgan-Daniel, Hannah F. Norton, Mary E. Edwards, Matthew Daley

**Affiliations:** 1 morgandanie.jane@ufl.edu, University of Florida Health Science Center Libraries, Gainesville, FL; 2 nortonh@ufl.edu, University of Florida Health Science Center Libraries, Gainesville, FL; 3 meedwards@ufl.edu, University of Florida Health Science Center Libraries, Gainesville, FL; 4 daleym@ufl.edu, University of Florida Health Science Center Libraries, Gainesville, FL

**Keywords:** Diversity, equity, inclusion, Inclusive language, Website, LibGuides, Signage, Academic Health Sciences Libraries

## Abstract

**Background:**

A Diversity, Equity, and Inclusion (DEI) Team at a university health science library created a checklist for inclusive language and conducted an assessment of their library's website, LibGuides, and physical and digital signage. Inclusive language was defined as “language that is free from words, phrases or tones that reflect prejudiced, stereotyped or discriminatory views of particular people or groups”.

**Case Presentation:**

The 32-item checklist facilitated the identification of gendered language, stereotypes, ableist language, racist language, stigmatizing language, slang, acronyms, and out-of-date terminology regarding physical and mental health conditions. From the library's website, 20 instances were noted for which improvements were necessary. Out of the 130 LibGuides reviewed, 23 LibGuides had no changes needed and 107 had changes identified relating to language inclusivity (14 strongly recommended changes and 116 suggested changes). Regarding the signage, one flyer was removed for reprinting.

**Conclusion:**

The checklist enabled the team to implement a number of improvements to the library's website and LibGuides. The checklist has been shared with Library Technology Services and the wider campus libraries' Usability Committee for future use, and has also been added to the DEI Team's LibGuide for use by others outside of the university.

## BACKGROUND

While academe has been attending to the need for diversity, equity, and inclusion (DEI) work in higher education for years, specific attention to divisive or discriminatory language on websites is more recent. The Black Lives Matter movement ignited a renewed effort to examine the impact of racism in all areas of life, including academic institutions [1, 2, 3]. Problematic language that is ableist, gendered, racist, or otherwise discriminatory or non-inclusive is an issue in health science libraries, as there are historical terms that are derogatory in both medicine and librarianship [4, 5]. These concerns are being addressed in part through efforts such as the National Library of Medicine's review of Medical Subject Headings (MeSH) terms and the National Institutes of Health's UNITE initiative to address structural racism [6, 7].

Scientific language is also rife with racist terms, as Jones points out particularly racially harmful terms that persist in STEM include the use of words like “master, slave, whitelist, blacklist, etc.” [8]. In STEM scholarly publications, emphasizing conscious language by focusing on writing that is free from bias has several benefits; in addition to making writing more respectful, it has the potential to improve the accuracy of writing and increase readership/audience by not excluding people with stereotypical or harmful descriptions or terminology [9].

Assessments of library websites and LibGuides typically focus on accessibility [10, 11, 12, 13], which, while important, should be expanded to include a broader review for non-inclusive language. While a handful of papers report evaluations of libraries' disability webpages (specifically, the existence of a disability page and information on that page), none were found in the published literature that conducted a thorough assessment of the library website or LibGuides across multiple dimensions of inclusion, including language. While librarians at the University of South Florida's Nelson Poynter Memorial Library undertook a similar assessment to ours, they focused on usability and accessibility [14].

The importance of avoiding outdated terminology and instead using welcoming and inclusive language on the website is reported by Brunskill [10], who interviewed students to learn what they wish to see on an accessibility page and how lack of that information impacts use of the library. Literature on evaluating the inclusivity of library signage is scarce, with articles focusing on signage amount, formatting, and location [15]. Although the importance of creating meaningful digital and physical signage is addressed by Polger and Stempler [16], they evaluate language inclusivity based on user-friendliness instead of DEI elements. Based on the research above regarding the importance of usability and accessibility in library physical and digital spaces as well as the impact of non-inclusive or discriminatory language, this website assessment project is essential to evaluate the state of language on library websites and suggest changes to increase usage of bias-free language. To ignore any of these issues could potentially impact usage, by situating the library as an unwelcoming organization that does not evaluate whether it is creating a safe physical and digital environment.

In order to fill this need for a holistic language inclusivity review, the University of Florida's Health Science Center Libraries (HSCL) created an extensive checklist for language inclusivity based on existing resources and implemented that list by reviewing all pages on their website, the library's LibGuides, and their digital and physical signage. The project's objectives were therefore to 1) create a checklist for inclusive language, 2) review the library's website, LibGuides, and signage using the checklist, 3) update these resources accordingly via language edits or additions, and 4) circulate the checklist to the university's other six libraries for potential use. The time it took to develop this thorough checklist benefits not only this library, but because the assessment tool is easily generalizable, it can also be a useful resource for other university libraries as well as libraries in other settings.

## CASE PRESENTATION

The HSCL serves six colleges; Dentistry, Medicine, Nursing, Pharmacy, Public Health and Health Professions, and Veterinary Medicine. To assess and improve the climate for DEI in the library, a team comprised of faculty and staff was created in 2018. This DEI team quickly began work establishing short and long-term goals and planning a variety of activities to assess and support a diverse and inclusive library climate [17]. Over the years since its formation, the DEI team has participated in numerous relevant training opportunities to provide team members with knowledge and skills in the areas needed to work on diversity and equity-related-projects [17].

Additionally, prior to starting many projects the team (or sub team) read related literature to get up to speed on any knowledge gaps. After completing survey-based climate assessments [18], the team moved on to a language assessment project as a subset of this larger goal.

Between 2020 and 2022, the DEI Team conducted a language inclusivity assessment of the HSCL's website [19], LibGuides [20], and physical and digital signage. The team defined inclusive language as “language that is free from words, phrases or tones that reflect prejudiced, stereotyped or discriminatory views of particular people or groups. It is also language that does not deliberately or inadvertently exclude people from feeling accepted” [21].

In March 2020, the team searched for existing literature relating to best practices for language inclusivity employed in library, health, or university settings. Nineteen useful resources were located through a general Google search and through the databases Library and Information Science Abstracts (ProQuest), Library, Information Science and Technology Abstracts (EBSCOHost), PubMed, and Web of Science [21, 22–39]. The team members individually reviewed assessments presented in these resources and each developed at least two questions for a draft checklist. A final version of the checklist was compiled after discussion, through which similar questions were removed and the remaining questions were grouped into identity-based themes (Appendix A). The final 32-item checklist covers topics including demographic information collected in webforms, identification of gendered language, stereotypes, ableist language, racist language, stigmatizing language, slang, acronyms, and out-of-date terminology regarding physical and mental health conditions. The checklist also includes an item addressing whether images on the website are reflective of the communities the library serves; while not strictly language-related, the team felt this significantly impacts inclusivity of communication through the website.

Using the checklist, the team began reviewing the 33 webpages of the library's website in June 2020, not including links to external webpages or HSCL's LibGuides at this stage of the project. Each webpage was assessed by one of five team members, who used a spreadsheet pre-populated with the checklist to record any language that needed to be changed [40]. Team members were asked to “Answer Yes, No, or N/A for each checklist question”. When changes needed to be made to a webpage, the reviewers were prompted to “note which page and quote the sentence that needs to be changed and why.” The team members also recorded any words or sentences that they were unsure of for discussion and made a note of DEI-related content that could potentially be added based on the language inclusivity literature consulted before beginning the assessment. Once each website page had been reviewed and data entered into the spreadsheet, the team met in late August 2020 to reach a consensus on corrections and potential additions. The library's website manager then implemented changes to the website in October 2020.

After reviewing the whole website, 20 instances were noted for which improvements needed to be made. One improvement involved adding pronouns for employees who opted in via response to an email invitation to contribute to a culture where gender identity is not assumed but instead affirmed. We also altered the website to refer to services for invisible disabilities, such as assistive technology available for converting text to voice, and added a section for sensory-friendly spaces on our “patrons with disabilities” page with information about nap pods and individual study rooms (sensory-friendly refers to consideration of environmental factors like light, sound, or smell that may contribute to sensory overload). Other changes included using student first language instead of faculty first to reduce implications of hierarchy or preferential treatment (for example, “the library provides research support for students and faculty”); spelling out acronyms; and removing outdated language with negative connotations such as “earmarked,” as this term has been associated with enslavement [41]. The team's recommendations for website content additions included creating a DEI statement for the library; developing a code of conduct for the library's online and physical spaces; and adding information about parking, assistive technologies, and emergency evacuation procedures to our “patrons with disabilities” page. There was one instance of a change being discussed but discarded due to concerns about historical accuracy, this was the word “chairman” which was found in an archives-related webpage.

In March 2021, the checklist was repurposed for reviewing HSCL's 130 LibGuides. As with the website review, each LibGuide was independently reviewed by one of five team members using a version of the checklist in Excel. While the checklist did not include items related to link-checking or general accessibility concerns, when team members found broken links or inaccessible formatting, this information was included among the recommendations for the guide author. Through group discussion, it was determined that 107 of the LibGuides had changes relating to language inclusivity (14 with “strongly recommended changes” and 116 with “suggested changes”) and that 23 LibGuides had “no changes needed.” Across all LibGuides, 186 different recommendations were made, in some cases with multiple recommendations made on a single guide. The project lead reached out to each LibGuide's primary editor to request that the “strongly recommended” changes be swiftly implemented, and that “suggested changes” be implemented if the editor agreed (see example email in Appendix B). Strongly recommended changes, defined as language that is harmful or offensive, included: altering the word “victim” to the more empowering term “survivor” in the description of a resource for intimate partner violence; capitalizing “Black” and “Indigenous Peoples,” as well as other names of nationalities, peoples, and cultures; changing an outdated National Library of Medicine classification of “Mental Retardation” to “Intellectual Disability;” adjusting the term “vulnerable populations” to “marginalized populations” as this more proactively calls attention to the role of social structures in the creation of health disparities; using “congenital anomalies” in place of “birth defects” due to negative terminology connotations identified by some populations; changing the word “slaves” to “enslaved people” in relation to a LibGuide on a past historical exhibit; and adding a disclaimer to another LibGuide to reflect that “women's health” is the terminology commonly used in medicine, but that the library recognizes that use of the word “women” in this context should be inclusive of all people with uteruses regardless of gender identity. Many suggested changes mirrored those identified during the website assessment such as spelling out abbreviations, adding pronouns in LibGuide profile boxes, and using student first instead of faculty first language. Suggested changes exclusively found in the LibGuides were altering “elderly” to “older adults,” adding quotation marks around gendered language used in the description associated with an external exhibition hosted by HSCL, and editing images of nuclear families to represent different types of families.

An assessment of signage and non-digital flyers and forms occurred via a walkthrough of the physical library by three team members in January 2022. One flyer on liaison services was pulled for reprinting, so that the language could be altered to student first instead of faculty first. The website manager then looked at files that were available for display on digital library signage but did not see any content that required modification for inclusive language.

## DISCUSSION

This article fills a gap in the literature by providing a concrete example of how to conduct an inclusive language review of a library website. The team found that using the checklist was an effective method for identifying potential improvements to our website and LibGuides. Members of the project group included HSCL's Chair and the library's website manager; this was an advantage because the team did not face any management-level barriers to implementing the website or LibGuide changes. After implementing the website changes, the team shared the checklist with Library Technology Services and the wider libraries' Usability Committee for future use, thus fulfilling the fourth project objective. The checklist was also added to the DEI Team LibGuide [42]. The team believes this process is generalizable outside of the University of Florida and make the checklist available here (Appendix A) so that it can be found and used beyond this specific context.

The larger changes to the website included creating a Code of Conduct, applicable to our physical and online spaces [43]; adding information on sensory-friendly spaces to our Patrons with Disabilities page [44]; and updating our floor maps with clearer information about stairs, elevators, gender-neutral restrooms, and our lactation pod [45]. A number of the recommendations for LibGuide authors mirrored findings from the website, with the most common being suggestions to add personal pronouns, use student first language, and spell out all abbreviations and acronyms (see Table 1 for additional details).

**Table 1:** Most common recommended changes for HSCL's website and LibGuides

Recommendation	# times noted on website	# times noted in LibGuides
Consider adding personal pronouns	1	65
Consider using student first language	7	46
Spell out all abbreviations and acronyms	4	20
Update or remove broken links	0	24
Change specific words or phrases (e.g., earmarked, victim)	3	14
General accessibility concerns (e.g., font size, color contrast, screen-reader capability)	0	8
Improve gender-inclusive language	0	9
Other	5	10
**Total number of recommendations**	**20**	**186**

While the team was empowered to make immediate changes to the website upon identifying areas for improvement, potential changes to LibGuides were sent to guide authors without further intervention from the team. Guide authors were largely appreciative of the review process and specific suggestions for their guides, though there was pushback from some individuals who were uninterested in sharing their own pronouns or using nongendered language in their LibGuides content. On reflection, the team became aware of media conversations that emphasize how sharing pronouns should not be mandatory in a workplace, in case some individuals do not feel comfortable or ready to share their gender identities [46]. The process of requesting changes to LibGuide language could be considered in the context of academic freedom; however, none of the guide authors explicitly mentioned this concern, the recommendations were made by a team of peers, and no consequences were imposed for noncompliance. This study had two limitations. While the checklist was developed collaboratively, it was not normed prior to use. Also, only one researcher evaluated each webpage and Libguide, which introduced the possibility of reviewer fatigue and confirmation bias.

Going forward, the team needs to determine how often an inclusive language review of LibGuides should be repeated, the process for reviewing newly-created guides, and how to follow-up with guide authors if changes are not made, particularly those that were strongly recommended. Other institutions may benefit from incorporating inclusive language recommendations into LibGuide creation standards or guidelines; however, the University of Florida's decentralized and largely un-mediated LibGuide administration does not make this feasible at our institution at this time. To improve understanding across the library about the inclusive language review process and increase buy-in, the team is considering providing a brief internal training on the checklist and offering badges for LibGuides that meet inclusive language standards. The usability of the checklist could be improved somewhat by further grouping like items together (e.g., all those related to gender inclusivity) and framing all items positively (so that a positive response to each would indicate a positive indicator of language inclusivity). The checklist will also need to be updated periodically as language continues to change and additional strategies are developed for inclusivity. Additionally, the team may engage with others across the University of Florida, who are beginning to consider broader LibGuides review in the model of the California State University Libraries' LibGuides Open Review Discussion Sessions (LORDS) Project, which goes beyond language and accessibility to address expanding race awareness and librarian positionality in LibGuides [47, 48].

Another remaining action item from the initial website review was to create a DEI statement to be posted on the HSCL website. In the past year, another subset of the HSCL DEI Team drafted an HSCL Vision and Values Statement, which was then reviewed, edited, and adopted by the HSCL staff as a whole. While the statement speaks broadly of the work done in our library, diversity, equity, and inclusion permeate the statement. The DEI Team has thus determined that, for the time being, a separate DEI statement is not needed [49].

Overall, the team found that using the checklist generated multiple improvements to HSCL's website and LibGuides. We therefore highly recommend that other libraries consider performing an inclusive language audit, with our checklist available as a starting point. Ideally, as in our organization, website managers will be aware of the importance of both accessibility and language inclusivity. In institutions where this is not the case, libraries should strive to raise awareness of language inclusivity as a DEI issue, not only for website managers but also amongst all library employees who are creating content for websites, LibGuides, and signage.

## Data Availability

Data associated with this article are available in the Open Science Framework at https://osf.io/f27wc/.

## SUPPLEMENTAL FILES

**Appendix A:** 32-item Inclusive Language Checklist

**Appendix B:** LibGuide Changes
