# Supplementary material for: Developing and conducting a language inclusivity assessment on a health science library's website, LibGuides, and signage
Source: J Med Libr Assoc. 2024 Jan 16;112(1):48–54. doi: 10.5195/jmla.2024.1691 (PMC11189139; doi:10.5195/jmla.2024.1691)
Supplement: Supplementary file 1 — Appendix A: 32-item Inclusive Language Checklist [file jmla-112-1-48-s01.pdf]

Appendix A:

32-item Inclusive Language Checklist

1. Does this webpage use gender neutral/gender-inclusive language? Examples: using they instead of he or she, using patrons/folks/friends instead of ladies and gentlemen, not using sir/madam.
2. Do team members share their pronouns on the website's contact pages? If pronouns are requested by website forms, are there gender-inclusive options such as they?
3. If any webpages or linked forms refer to or ask about gender identity, is this necessary? If it is necessary, for example if conducting a diversity-related survey, are additional options provided to man or woman, at minimum with an option to self-describe?
4. If the webpage refers to restrooms, does it indicate the location of gender-inclusive restrooms, family restrooms, or lactation rooms (if there are any)?
5. Do any webpages or linked forms ask for information about race or ethnicity? If so, is this necessary and is the list alphabetical? For example, white should not be at the top of the list. Are there options to check more than one race or ethnicity category?
6. If asking someone's religion or referring to holiday opening hours, does the webpage use more general terms like winter break instead of Christmas break? Is Christianity listed first in any lists of religions when lists should be alphabetical instead?
7. If any webpages or linked forms refer to or ask about marital status, is this necessary? If so, are the words spouse, partner, or significant other used instead of the words husband or wife? If parental status is mentioned, is the word parent(s) used instead of Mom and Dad? Ensure that the webpage avoids assumptions relating to children having two parents, two parents who are married, or spouses being of the opposite sex.
8. If any career-related language is used, is it gender neutral? E.g. instead of the word policeman use police officer, unless it's someone's official title.

9. Does the webpage avoid stereotyping population groups? Ensure that no personal assumptions are made based on gender, culture, ancestry, age, or other identities, e.g., mom doing housework or girls wearing make-up.
10. Does the webpage avoid using any ableist language or out-of-date terminology for people with disabilities? For example, remove any uses of the word handicapped.
11. If the website does mention disabilities, does it use people-first language? For example, the website should say “person with a disability” instead of “disabled person”. Note – some groups of people use identity-first language, so disability-related language should be changed if user feedback is received.
12. Are invisible disabilities recognized or does the webpage only refer to physical disabilities?
13. Is information prominently displayed on the webpage about services for people with disabilities? E.g., ADA-compliant accessible tables.
14. Are responses to any website form submissions gender neutral? If the patron does not use a gender-specific identifier, ensure the response does not use gendered language either. E.g., avoid the use of “Dear Sir/Madam” or “Dear Mr. [last name]. Instead, use “Dear [first name last name]”.
15. If sexual orientation is mentioned, is this necessary? If so, is the list alphabetical? Ensure that there are a number of options provided, encompassing at a minimum: Asexual or Ace, Bisexual, Gay, Heterosexual, Lesbian, Pansexual, Queer.
16. If mental health language is included, does the website avoid the use of any out-of-date, derogatory, or stigmatizing terms?
17. Does the website have a DEI statement on the front page or prominently displayed?
18. If any policies are listed on the website do they avoid the use of gendered language and stereotypes e.g., in relation to dress codes?

19. Are the photos and pictures on the website reflective of the communities that are served? For example, a mixture of pictures is ideal, where possible encompassing all genders, non-white people, people who are disabled etc.
20. Do forms reflect that people can use the name that they generally go by rather than their legal name? (This is often very important for people who identify as transgender).
21. Are the options for honorifics in forms gender-inclusive? This should include an opt-out option and a self-describe option, in addition to Dr., Ms., Mr., Mx. at a minimum.
22. Does the webpage avoid using slang or jargon?
23. Are the proper names of nationalities, peoples, and cultures capitalized? For example, use Black not black.
24. Is “first name” used instead of “Christian name”?
25. Does the webpage avoid language that implies weakness or infirmity? For example, do not use the phrase “wheelchair-bound”, but instead say, “the employee uses a wheelchair”.
26. If age is mentioned, does the webpage avoid implying that a particular age group is more or less able? Note – remember that the words “older” and “younger” are context-specific, so should only be used if clearly described and very necessary.
27. If age is mentioned, does the webpage avoid the use of the word elderly? The term “elderly” can have stereotypical connotations. An alternative phrase is “people over the age of 65”.
28. Does the webpage avoid phrases that equate “thin” or “able-bodied” with health?
29. Does the webpage avoid the use of terms that reinforce stigma, imply helplessness, or invite pity? For example, use the phrase “person with AIDS” instead of “AIDS victim”.
30. Are all acronyms spelled out once on each page?

31. Is student-first language used? E.g., student, staff, and faculty.
32. Is “undocumented” used instead of “illegal immigrant” or “illegal”?
