# Supplementary material for: Developing and conducting a language inclusivity assessment on a health science library's website, LibGuides, and signage
Source: J Med Libr Assoc. 2024 Jan 16;112(1):48–54. doi: 10.5195/jmla.2024.1691 (PMC11189139; doi:10.5195/jmla.2024.1691)
Supplement: Supplementary file 2 — Appendix B: LibGuide Changes [file jmla-112-1-48-s02.pdf]

Appendix B:

Sample email to colleagues with recommendations for LibGuide changes

Hi Ariel, Hannah, Mary, and Nina,

Over the last few months the HSCL DEI Team has been reviewing HSCL LibGuides for language inclusivity using a checklist that we created (we can share it with you if you're interested). We came up with three categories for the LibGuides; no changes needed; suggest changing; and strongly recommend changing.

At the time of reviewing your LibGuides, we noticed the following, and are hoping you'd consider making some LibGuide updates.

Please let us know if you have any questions!

List of LibGuide(s) alongside potential changes

Information Resources for Medical Students: https://guides.uflib.ufl.edu/uf-medical-students

Strongly suggest changing:

- Not all proper names of nationalities, peoples, and cultures are capitalized, please check the racism in medicine tab and change in book descriptions
- On Information for Your Patients tab there's a description of Peaceful Paths describing domestic violence survivors as victims. Checked domestic violence organizations and they recommend using the word survivor as it is considered more empowering. Sometimes victim/survivor is used

Suggest changing:

- Pronouns could be added (instructions for editing profile boxes are viewable here)
- Could add information about services for patrons with disabilities or link to HSCL webpage https://library.health.ufl.edu/services/patrons-with-disabilities/
- Uses the term elderly on health literacy tab, suggest changing to older adults
- Health Literacy is not spelled out on health literacy tab, suggest removing acronym
- On writing and citing tab, suggest changing to "student first" language e.g. student, staff, and faculty
- Grammar error note on first page: This guide is designed to be a central resource to help you find the materials you need related throughout your time as a medical student.
- Link error: VPN software link on first page just links to Smathers general page
- Link error: in Writing and Citing Tab, the Purdue links don't link to the specific pages stated
- Link error: Emed Tv link not working on Information for Your Patient tab
- Grammar error on health literacy page - not seek care until it an emergency; or use emergency services for minor conditions
- Realm link not working on health literacy tab: http://www.adultmeducation.com/assessmenttools_1.html

- Link not working on Mobile Resources page: http://http//guides.uflib.ufl.edu/mobilehealth
- Links not working under "specific journals": https://libraries.flvc.org/sunset
- Disability misspelled and living misspelled in Health Outcomes and Policy tab

Thank you,

Jane, Hannah, Mary, and Matthew (on behalf of the DEI Team)
